# Reverse Dispersion Entropy: A New Complexity Measure for Sensor Signal

**DOI:** 10.3390/s19235203

**Published:** 2019-11-27

**Authors:** Yuxing Li, Xiang Gao, Long Wang

**Affiliations:** 1 School of Automation and Information Engineering, Xi’an University of Technology, Xi’an 710048, China; gaoxiang@xaut.edu.cn; 2 School of Marine Science and Technology, Northwestern Polytechnical University, Xi’an 710072, China; wanglongwl@mail.nwpu.edu.cn

**Keywords:** permutation entropy (PE), weighted-permutation entropy (W-PE), reverse permutation entropy (RPE), reverse dispersion entropy (RDE), time series analysis, complexity, sensor signal

## Abstract

Permutation entropy (PE), as one of the powerful complexity measures for analyzing time series, has advantages of easy implementation and high efficiency. In order to improve the performance of PE, some improved PE methods have been proposed through introducing amplitude information and distance information in recent years. Weighted-permutation entropy (W-PE) weight each arrangement pattern by using variance information, which has good robustness and stability in the case of high noise level and can extract complexity information from data with spike feature or abrupt amplitude change. Dispersion entropy (DE) introduces amplitude information by using the normal cumulative distribution function (NCDF); it not only can detect the change of simultaneous frequency and amplitude, but also is superior to the PE method in distinguishing different data sets. Reverse permutation entropy (RPE) is defined as the distance to white noise in the opposite trend with PE and W-PE, which has high stability for time series with varying lengths. To further improve the performance of PE, we propose a new complexity measure for analyzing time series, and term it as reverse dispersion entropy (RDE). RDE takes PE as its theoretical basis and combines the advantages of DE and RPE by introducing amplitude information and distance information. Simulation experiments were carried out on simulated and sensor signals, including mutation signal detection under different parameters, noise robustness testing, stability testing under different signal-to-noise ratios (SNRs), and distinguishing real data for different kinds of ships and faults. The experimental results show, compared with PE, W-PE, RPE, and DE, that RDE has better performance in detecting abrupt signal and noise robustness testing, and has better stability for simulated and sensor signal. Moreover, it also shows higher distinguishing ability than the other four kinds of PE for sensor signals.

## 1. Introduction

Due to the continuous development of measurement technology and the constant updating of high sensitivity sensor equipment, the accuracy of measured time series is greatly improved, which is conducive to the further analysis and processing of time series [1,2]. The complexity of time series is one of the most important means to represent the characteristics of time series. Entropy, as an effective complexity measure of time series, has been widely developed and used in different fields. Classic examples include permutation entropy (PE) [3], sample entropy (SE) [4], approximate entropy (AE) [5], fuzzy entropy (FE) [6], and multi-scale ones. However, among these different kinds of entropy, PE has successfully attracted attention from academics and practitioners by virtue of its own advantages.

In 2002, PE was first suggested in a scientific article by Bandt and Pompe [7]. As a complexity measure, PE introduced arrangement into time series, and determined each arrangement pattern according to the neighboring values. PE has the characteristics of easy implementation and high computation efficiency. With its own advantages, PE has been widely used in different fields, including the medical field [8], mechanical engineering field [9,10], economic field [11,12], and underwater acoustic field [13,14]. Aiming at weaknesses of PE, many revised PE methods have been proposed to improve the performance of traditional PE.

In 2013, Fadlallah et al. brought forward weighted-permutation entropy (W-PE) and first applied it to electroencephalogram signal processing [15]. In order to solve the limitation of PE, W-PE introduced amplitude information to weight each arrangement pattern by using variance information. Compared with PE, W-PE responds better to the sudden change of amplitude, in addition, it has better robustness and stability than PE at low signal-to-noise ratio (SNR). As an improvement of PE, W-PE has important influence and status in different fields [16,17,18]. For example, W-PE can show a better performance than PE in distinguishing Alzheimer’s disease patients from normal controls [19].

In 2016, Rostaghi and Azami proposed dispersion entropy (DE) to quantify the complexity of time series and first applied it to electroencephalograms and bearing fault diagnosis database [20]. Unlike W-PE, DE introduced amplitude information to map the original signal to the dispersion signal by using the normal cumulative distribution function (NCDF). Compared with PE, DE has a better ability to detect the change of simultaneous frequency and amplitude, and also has a better ability to distinguish different datasets and requires less computation time. In [21], PE, AE, and DE were compared, the results suggest that DE leads to more stable results in describing the state of rotating machinery, and it is more suitable for real-time applications.

In 2017, reverse PE (RPE) was put forward by Bandt and employed to identify different sleep stages by using electroencephalogram data [22]. Since RPE is defined as the distance from white noise, it has the opposite trend with PE, W-PE, and DE. In [23,24], RPE was used for feature extraction of underwater acoustic signals, compared with PE, RPE has more stable performance and a higher classification recognition rate.

To improve the performance of PE and integrate the advantages of DE and RPE, we propose a new complexity measure for analyzing time series in this paper, and term reverse dispersion entropy (RDE) through introducing amplitude information of DE and distance information of RPE. In the next section, RDE is described in detail through comparison with PE, W-PE, RPE, and DE. In Section 3 and Section 4, simulation experiments are carried out to further compare and analyze five kinds of PE. Finally, we summarize the total research work in Section 5.

## 2. Reverse Dispersion Entropy

**Figure 1:**
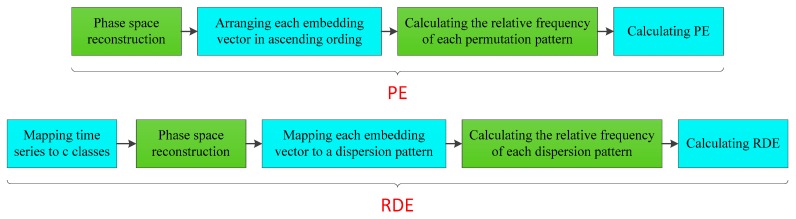
The flow chart of permutation entropy (PE) and reverse dispersion entropy (RDE).

RDE, as a new complexity measure for analyzing time series, takes PE as its theoretical basis and combines the advantages of DE and RPE. The flow chart of PE and RDE are shown in Figure 1. As shown in Figure 1, all steps of PE and RDE are different except for phase space reconstruction.

The specific steps of RDE and detailed comparisons with the other four entropies are as follows [7,15,20,22]:

Step 1: mapping time series to c classes.

(1) Mapping by normal cumulative distribution function (NCDF)

For a time series X={x(i), i=1, 2, ⋯, T} with T values, we map X to Y={y(i), i=1, 2, ⋯, T} by NCDF, where y(i) ranges from 0 to 1.

(2) Mapping by round(c.y(i)+0.5).

We map Y to Z={z(i), i=1, 2, ⋯, T} by using round(c.y(i)+0.5), where c is the number of classes and z(i) is a positive integer from 1 to c. There is no difference in this step between DE and RDE.

Step 2: phase space reconstruction.

We reconstruct Z into L. embedding vectors with the time delay τ and embedding dimension m, respectively. The matrix consisting of all embedding vectors can be represented as follows:

(1) $$\left[\begin{array}{c} \left\{z\left(1\right),\;z\left(1+\tau \right),\;\cdots ,\;z\left(1+\left(m-1\right)\tau \right)\right\}\\ \begin{array}{ccc} \begin{array}{ccc} \begin{array}{ccc} \begin{array}{ccc} & & \vdots \end{array} & & \end{array} & & \vdots \end{array} & & \end{array}\\ \left\{z\left(j\right),\;z\left(j+\tau \right),\;\cdots ,\;z\left(j+\left(m-1\right)\tau \right)\right\}\\ \begin{array}{ccc} \begin{array}{ccc} \begin{array}{ccc} \begin{array}{ccc} & & \vdots \end{array} & & \end{array} & & \vdots \end{array} & & \end{array}\\ \left\{z\left(L\right),\;z\left(L+\tau \right),\;\cdots ,\;z\left(L+\left(m-1\right)\tau \right)\right\} \end{array}\right]$$

where the number of embedding vectors L is equal to T−(m−1)τ. There is no difference in this step between PE, W-PE, RPE, and RDE.

Step 3: mapping each embedding vector to a dispersion pattern.

Since the embedding dimension and the number of classes are m and c, respectively, there exists $c^{m}$ dispersion patterns, and each embedding vector can be mapped to a dispersion pattern π. For PE and W-PE, there exist m! arrangement patterns, which is different from DE and RDE. However, there is also no difference in this step between DE and RDE.

Step 4: calculating the relative frequency of each dispersion pattern.

The relative frequency of i-th dispersion pattern can be expressed as follows:

(2) $$P\left(\pi_{i}\right)=\frac{Number\left\{\pi_{i}\right\}}{N-\left(m-1\right)\tau }\;\left(1\leq i\leq c^{m}\right)$$

In truth, $P\left(\pi_{i}\right)$ represents the proportion of the number of i-th dispersion patterns to the number of embedding vectors. The four kinds of entropy are the same in this step.

Step 5: calculating RDE.

Like RPE, RDE is defined as the distance to white noise by combining distance information. It can be expressed as:

(3) $$H_{RDE}\left(X,m,c,\tau \right)=\overset{c^{m}}{\underset{i=1}{\sum }}{\left(P\left(\pi_{i}\right)-\frac{1}{c^{m}}\right)}^{2}=\overset{c^{m}}{\underset{i=1}{\sum }}P{\left(\pi_{i}\right)}^{2}-\frac{1}{c^{m}}$$

when $P\left(\pi_{i}\right)=1/c^{m}$, the value of $H_{RDE}\left(X,m,c,\tau \right)$ is 0 (minimum value). In step 5, the calculation formulas of PE, W-PE, and DE are the same based on the definition of Shannon entropy, however, the calculation formula of RDE is the same as that of RPE by combining distance information.

When there is only one dispersion pattern, that is $P\left(\pi_{i}\right)=1$, the value of $H_{RDE}\left(X,m,c,\tau \right)$ is $1-\frac{1}{c^{m}}$ (maximum value). Therefore, the normalized RDE can be expressed as:

(4) $$H_{RDE}=\frac{H_{RDE}\left(X,m,c,\tau \right)}{1-\frac{1}{c^{m}}}$$

Based on the test of simulation signals and real sensor signals, the recommended parameters of RDE are shown in Table 1. More details about PE, W-PE, DE, and RPE can be found in [7,15,20,22].

## 3. Simulations with Synthetic Signals

### 3.1. Simulation 1

**Figure 2:**
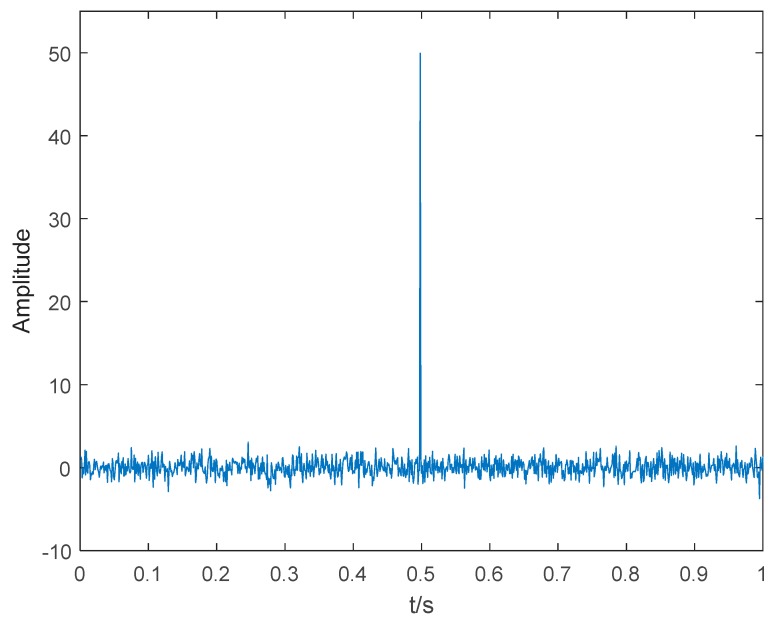
The time domain waveform of *y*.

**Figure 3:**
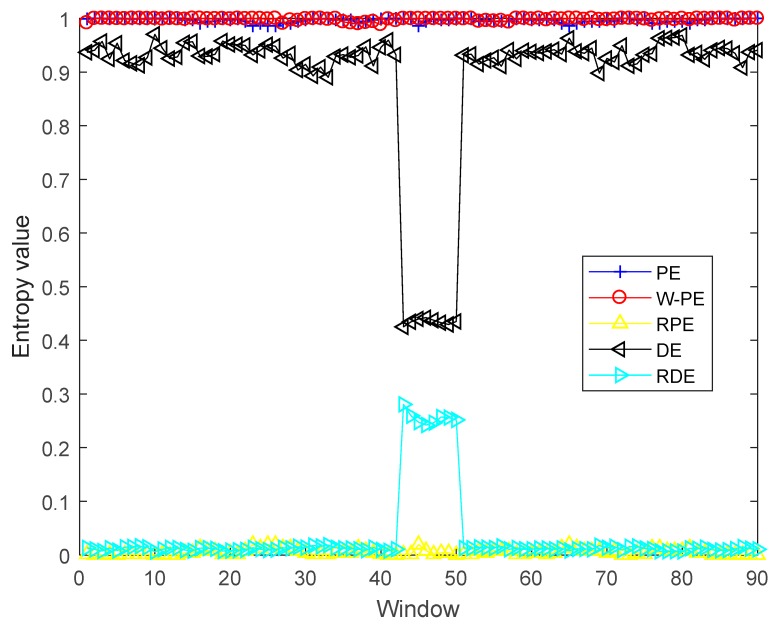
The five entropies of *y*. W-PE: weighted-permutation entropy; RPE: reverse permutation entropy; DE: dispersion entropy.

To demonstrate the ability of RDE to detect mutation signals, we carried out a simulation experiment similar to [15]. The synthetic signals are as follows:

(5) $$\left\{ \begin{array}{l} y=x+s\\ x=\left\{ \begin{array}{l} 50,\;(t=0.498)\\ 0,\;(t>=0\&t<=1) \end{array}\right.\\ s=randn(t) \end{array}\right.$$

where the synthetic signal y with the sampling frequency of 1 kHz is composed of white Gaussian noise s and impulse signal x. The time domain waveform of y is shown in Figure 2. Five entropies are calculated by using a sliding window of 80 samples with 70 overlapped ones. For DE and RDE, the parameter c is 6. For all five entropies, the embedding dimension and time delay are 2 and 1. The five entropies of y are shown in Figure 3. Table 2 shows 5 entropies in the windows from 42 to 51. As shown in Figure 3 and Table 2, when the windows contain the impulse signal, the values of DE and RDE have a significant decrease and increase. For further comparison, the means of the five entropies and their variation ratios are shown in Table 3. A is the means of 82 windows without an impulse signal, B is the means of 8 windows with an impulse signal, and the variation ratio is the ratio of maximum to minimum of A and B. As shown in Table 3, for PE, W-PE, and RPE, A and B are very close, and the variation ratios are from 1.0002 to 1.04; for DE and RDE, there are obvious differences between A and B, the variation ratios are obviously greater than 1; DE has a variation ratio of 2.1503, and RDE has a variation ratio of up to 21.8034. The simulation results show that DE and RDE can detect mutation signals, and RDE with the highest variation ratio has better performance than the other four entropies in detecting mutation signals.

### 3.2. Simulation 2

**Figure 4:**
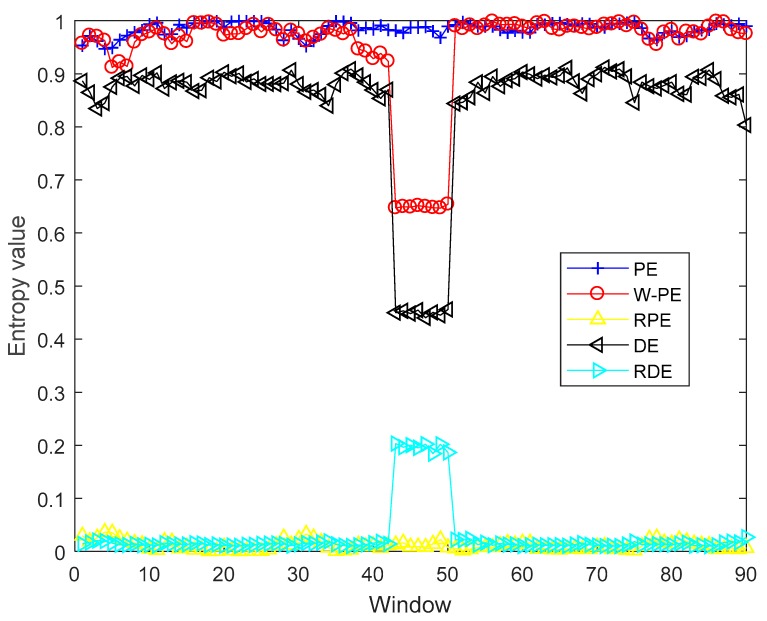
The five entropies of *y*.

Based on the recommended parameters range of RDE, we changed the embedding dimension of simulation 1 to 3. In view of $T>c^{m}$ and the sliding window of 80 samples, c can be set to four. Like simulation 1, simulation 2 was carried out with different parameters from simulation 1. The five entropies of y are shown in Figure 4. As shown in Figure 4, when the windows contain the impulse signal, the values of W-PE and DE have a significant decrease, and the values of RDE have a dramatical increase. Unlike simulation 1, W-PE with the embedding dimension of three can detect the mutation signal, the simulation results show that the value of embedding dimension can affect the capability of W-PE in detecting mutation signals.

Like Table 3, the means of the five entropies and their variation ratios are shown in Table 4. A is the means of 82 windows without an impulse signal, B is the means of eight windows with an impulse signal, and the variation ratio is the ratio of maximum to minimum of A and B. As shown in Table 4, for PE and RPE, A and B are close, and the variation ratios are almost 1 (1.0021 and 1.1468, respectively); for W-PE and DE, there are obvious differences between A and B, the variation ratios are obviously greater than 1 and less than 2 (1.5037 and 1.958, respectively); RDE has a variation ratio of up to 14.0143. The simulation results show that W-PE can detect mutation signals with the embedding dimension of three, DE and RDE can also detect mutation signals under different parameters, and RDE with the highest variation ratio have better performance than other four entropies in detecting mutation signal.

### 3.3. Simulation 3

In order to verify the robustness of RDE to noise, we carried out noise robustness testing by using the same impulse signal x in simulation 1, and the synthetic signals *y* with different SNRs can be obtained by adding white Gaussian noise to *x*. All parameters are consistent with simulation 1. We set the embedding dimension and time delay to three and one for the five entropies and set c to six for DE and RDE.

**Figure 5:**
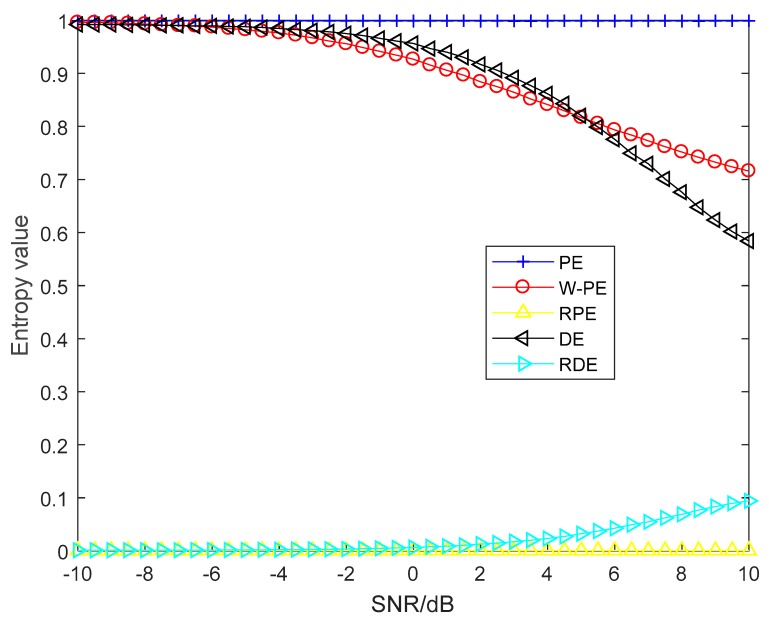
The five entropies of synthetic signal under different signal-to-noise ratios (SNRs).

The five entropies of synthetic signal under different SNRs from −10 dB to 10 dB are shown in Figure 5, and each entropy value is the mean under 1000 calculations. As shown in Figure 5, the values of PE and RPE have barely changed for different SNRs and are close to 1 and 0, respectively; the values of W-PE and DE monotonically decrease with the increase of SNR; the values of RDE monotonically increase with the increase of SNR. For further comparison of W-PE, DE, and RDE, the three entropies under −10 dB and 10 dB and their variation ratios are shown in Table 5. A and B are the entropies under 10 dB and −10 dB, and Max(A,B)/Min(A,B) is the ratio of maximum to minimum of A and B. As shown in Table 5, for W-PE and RDE, there are differences between A and B, the variation ratios are 1.3909 and 1.6993, respectively; RDE has a variation ratio of up to 94.3. Therefore, RDE can better reflect the difference under different SNRs than the other four entropies. The simulation results show that RDE with the highest variation ratio has better robustness to noise than the other four entropies.

### 3.4. Simulation 4

**Figure 6:**
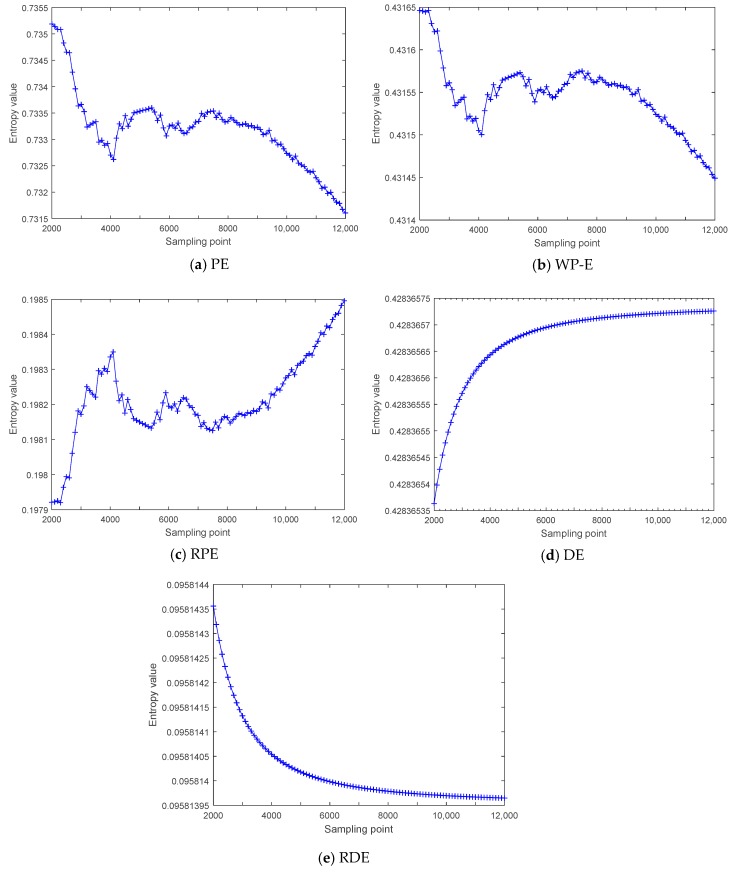
The five entropies of cosine signal with the frequency of 100 Hz.

In order to prove the stability of RDE for synthetic signal, we carried out stability testing by using the cosine signal of different lengths with the frequency of 100 Hz. For the five entropies, we set the embedding dimension and time delay to three and one and set c to six for DE and RDE. The five entropies of cosine signal with the frequency of 100 Hz are shown in Figure 6, the initial data length is 2000 sampling points, and 100 sampling points are added each time until the data length reached 12,000 sampling points.

As shown in Figure 6, the five entropies change in varying degrees with the increase of data length; the values of W-PE are from 0.43145 to 0.43165, the variation ranges of W-PE (10-4) are one order of magnitude lower than ones of PE (10-3) and RPE (10-3); the values of DE are from 0.4283653 to 0.4283657, the values of RDE are from 0.095814 to 0.0958144, and the variation ranges of DE ((10-7) and RDE (10-7) are smaller than ones of W-PE. The mean and standard deviation of five entropies for the cosine signal of different lengths are shown in Table 6. As shown in Table 6, RDE has the smallest standard deviation compared with the other four entropies. The stability testing results indicate that DE and RDE have better stability than the other three entropies under different length data.

### 3.5. Simulation 5

**Figure 7:**
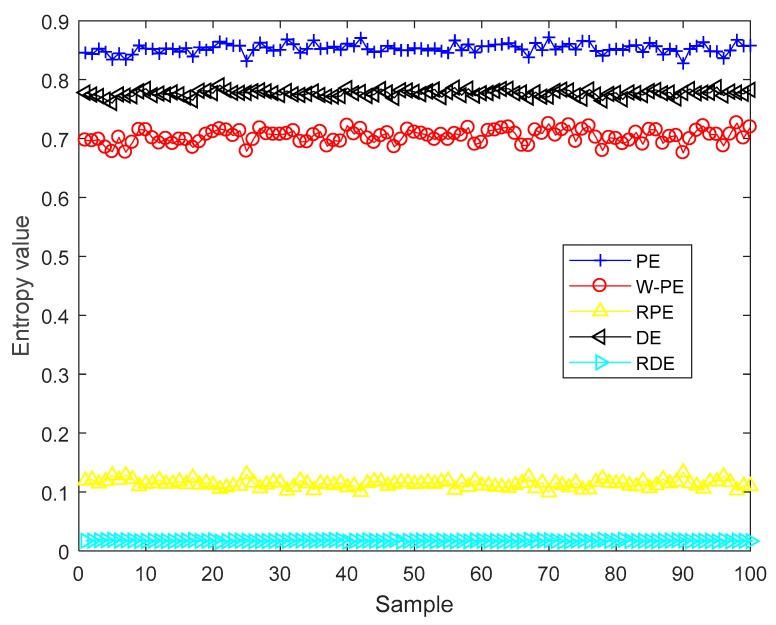
The five entropies of cosine signal under 10 dB.

In order to prove the stability of RDE for synthetic signal, we carried out stability testing by using the cosine signal cos(200πt) under 10 dB. The data length of each sample is 2000, we calculated the 5 entropies of 100 samples. The five entropies of cosine signal under 10 dB are shown in Figure 7. As shown in Figure 7, the entropy values of the same category are at the same level and have very little difference.

**Figure 8:**
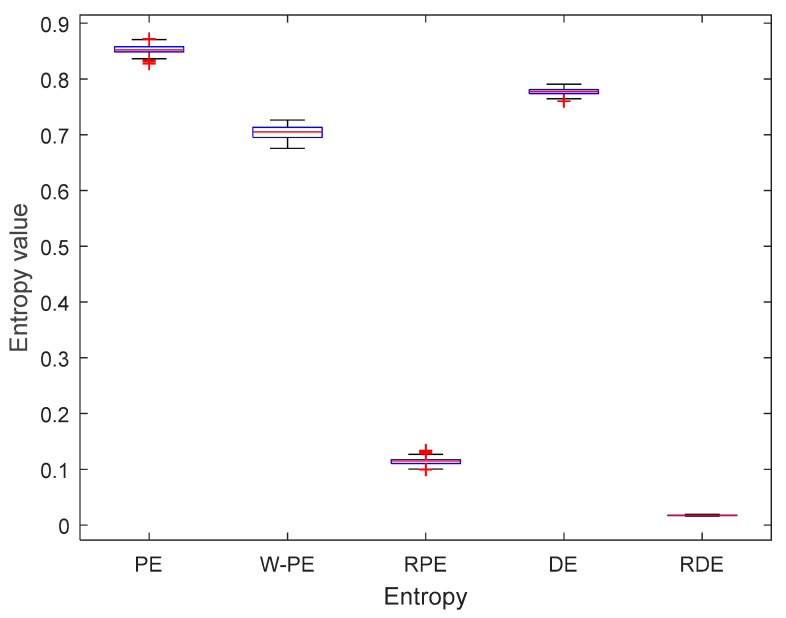
The complexity feature boxplots of five entropies for cosine signal under 10 dB.

To more intuitively compare the stability of the five entropies, the complexity feature boxplots of five entropies for cosine signal under 10 dB are shown in Figure 8. As shown in Figure 8, PE, W-PE, RPE, and DE have obvious fluctuations, however, RDE has the smallest fluctuation range compared to the other four entropies. The mean and standard deviation of the five entropies for the cosine signal under 10 dB are shown in Table 7. As shown in Table 7, RDE has the smallest standard deviation compared with the other four entropies. The experimental results show that RDE has better stability than the other four entropies under noisy conditions.

## 4. Application for Real Sensor Signals

### 4.1. Simulation 1

**Figure 9:**
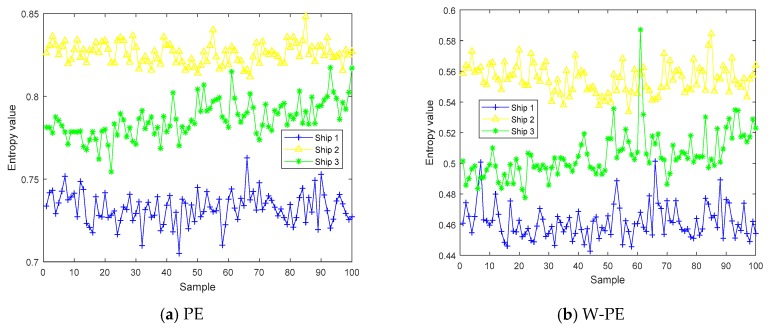

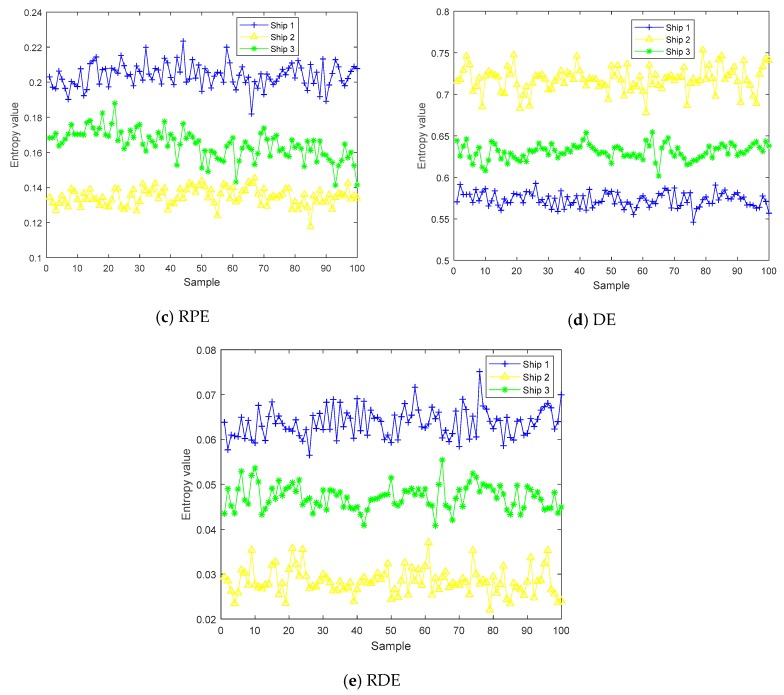
The five entropy distributions for three kinds of ship.

In order to compare the ability of five entropies to distinguish real sensor signals, we carried out complexity testing by using three kinds of ship signals, termed as ship 1, ship 2, and ship 3. Each sample was 5000 points with a sampling frequency of 44.1 kHz. The five entropy distributions of three kinds of ship are shown in Figure 9, and each kind of ship includes 100 samples. As shown in Figure 9, compared with the distributions of PE, W-PE, and RPE, the distributions of DE and RDE were easier to distinguish the three kinds of ship signals.

**Figure 10:**
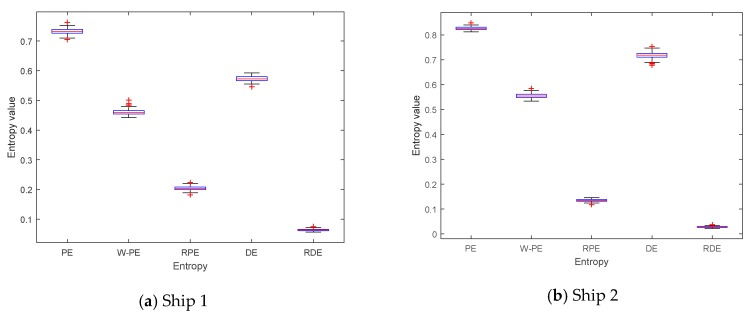

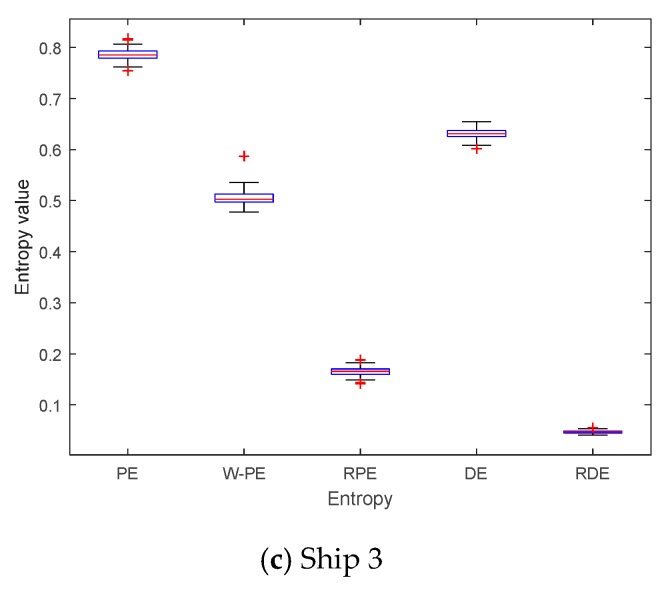
The complexity feature boxplots of five entropies for three kinds of ship.

The complexity feature boxplots of five entropies for three kinds of ship are shown in Figure 10, and the mean and standard deviation of five entropies for three kinds of ship are shown in Table 8. As shown in Figure 10 and Table 8, compared with the other four entropies, RDE had the smallest fluctuation range and standard deviation for each ship signal. The experimental results show that RDE has better stability for ship signals.

To further prove the distinguishing ability of RDE, we used a support vector machine (SVM) to distinguish the three kinds of ship signals; the classification results by five entropies for three kinds of ship are shown in Table 9. As seen in Table 9, PE and W-PE have a recognition rate of less than 95%; DE and RPE have a recognition rate of more than 95%; RDE has the highest recognition rate of up to 99%. The experimental results show that RDE has better distinguishing ability for ship signals.

### 4.2. Simulation 2

Like simulation 1 in Section 4.1, we carried out complexity testing by using three kinds of rolling bearings signals, termed as fault 1, fault 2, and fault 3, which come from the Case Western Reverse Laboratory [25]. Each sample is 2000 points with a sampling frequency of 12 kHz. The mean and standard deviation of the five entropies for three kinds of fault are shown in Table 10, and each kind of fault includes 50 samples. As shown in Table 10, for PE, W-PPE, RPE, and DE, the mean values of fault 1 and fault 2 are very close, which makes it difficult to distinguish the two faults; for RDE, there are obvious differences in the mean values of the three faults, and it has the smallest standard deviation compared to the other four entropies. The experimental results show that RDE has better stability for rolling bearing signals.

To further prove the distinguishing ability of RDE for rolling bearing signals, we used an SVM to distinguish three kinds of rolling bearing signals; the classification results by five entropies for three kinds of rolling bearing signals are in Table 11. As seen in Table 11, PE and W-PE have a recognition rate of less than 80%; RPE has a recognition rate of more than 80%; DE and RDE have a recognition rate of less than 95%; RDE has the highest recognition rate of up to 100%. The experimental results show that RDE has better distinguishing ability for rolling bearing signals.

## 5. Conclusions

This paper proposed a new complexity measure for analyzing time series and termed RDE. A large number of simulation experiments was carried out to verify the effectiveness of this complexity measure. Its main contributions are as follows:

1. Compared with PE, W-PE, RPE, and DE, RDE had better performance in detecting mutation signals under different embedding dimensions.
2. Compared with PE, W-PE, RPE, and DE, RDE had better robustness to noise and also better stability in the case of different length data and the presence of noise.
3. For real signals, RDE had better distinguishing ability and stability than PE, W-PE, RPE, and DE.

Overall, as an effective complexity metric, RDE could be used to analyze more real sensor signals in different fields.

## Figures and Tables

**Table 1:** The recommended parameters of RDE.

Parameters	τ	m	c	T
Values	1	2, 3	4, 5, 6, 7, 8	$T>c^{m}$

**Table 2:** The 5 entropies in the windows from 42 to 51.

Window	42	43	44	45	46	47	48	49	50	51
PE	0.995	0.993	0.995	0.997	0.995	0.996	0.997	0.998	0.997	0.996
W-PE	0.998	0.999	0.998	1.000	0.999	0.998	1.000	0.998	0.999	0.997
RPE	0.005	0.006	0.005	0.005	0.004	0.005	0.006	0.005	0.005	0.006
DE	0.934	0.432	0.434	0.435	0.436	0.435	0.433	0.434	0.436	0.935
RDE	0.012	0.265	0.257	0.252	0.248	0.252	0.256	0.256	0.255	0.011

**Table 3:** The means of the five entropies and their variation ratios.

Parameters	PE	W-PE	RPE	DE	RDE
A (Means of 82 windows)	0.9962	0.9980	0.0052	0.9345	0.0117
B (Means of 8 windows)	0.9964	0.9995	0.0050	0.4346	0.2551
Max(A,B)/Min(A,B)	1.0002	1.0015	1.0400	2.1503	21.8034

**Table 4:** The means of the five entropies and their variation ratios.

Parameters	PE	W-PE	RPE	DE	RDE
A (Means of 82 windows)	0.9844	0.9765	0.0109	0.8805	0.0140
B (Means of 8 windows)	0.9823	0.6494	0.0125	0.4497	0.1962
Max(A,B)/Min(A,B)	1.0021	1.5037	1.1468	1.9580	14.0143

**Table 5:** The three entropies under −10 dB and 10 dB and their variation ratios.

Parameters	W-PE	DE	RDE
A (10 dB)	0.7160	0.5839	0.0943
B (−10 dB)	0.9959	0.9922	0.0010
Max(A,B)/Min(A,B)	1.3909	1.6993	94.3000

**Table 6:** The mean and standard deviation of five entropies for the cosine signal of different lengths.

Parameters	PE	W-PE	RPE	DE	RDE
mean value	0.7334	0.4316	0.1982	0.4284	0.0958
standard deviation	$5\times 10^{-4}$	$3\times 10^{-5}$	$9\times 10^{-5}$	$9\times 10^{-8}$	$8\times 10^{-8}$

**Table 7:** The mean and standard deviation of five entropies for the cosine signal under 10 dB.

Parameters	PE	W-PE	RPE	DE	RDE
mean value	0.8510	0.7034	0.1154	0.7770	0.0173
standard deviation	0.0073	0.0113	0.0056	0.0048	0.0006

**Table 8:** The mean and standard deviation of five entropies for three kinds of ship.

	PE	W-PE	RPE	DE	RDE
mean value of ship 1	0.7325	0.4617	0.2039	0.5731	0.0637
standard deviation of ship 1	0.0096	0.0111	0.0069	0.0088	0.0033
mean value of ship 2	0.8259	0.5550	0.1347	0.7179	0.0284
standard deviation of ship 2	0.0065	0.0096	0.0049	0.0150	0.0030
mean value of ship 3	0.7862	0.5051	0.1646	0.6308	0.0472
standard deviation of ship 3	0.0112	0.0150	0.0083	0.0094	0.0028

**Table 9:** The classification results by five entropies for three kinds of ship.

PE	W-PE	RPE	DE	RDE
92.67%	93.33%	96%	98.33%	99%

**Table 10:** The mean and standard deviation of five entropies for three kinds of fault.

	PE	W-PE	RPE	DE	RDE
mean value of fault 1	0.7752	0.4932	0.1785	0.7378	0.0221
standard deviation of fault 1	0.0063	0.0040	0.0054	0.0045	0.0009
mean value of fault 2	0.9702	0.8820	0.0226	0.9480	0.0023
standard deviation of fault 2	0.0023	0.0069	0.0018	0.0028	0.0001
mean value of fault 3	0.9717	0.9045	0.0214	0.9227	0.0039
standard deviation of fault 3	0.0024	0.0073	0.0018	0.0135	0.0009

**Table 11:** The classification results by five entropies for three kinds of rolling bearing signals.

PE	W-PE	RPE	DE	RDE
74.67%	77.33%	83.33%	96.67%	100%
